# Performance of diffusion and perfusion MRI in evaluating primary central nervous system lymphomas of different locations

**DOI:** 10.1186/s12880-020-00462-7

**Published:** 2020-06-09

**Authors:** Zhen Xing, Nannan Kang, Yu Lin, Xiaofang Zhou, Zebin Xiao, Dairong Cao

**Affiliations:** 1 Department of Radiology, First Affiliated Hospital of Fujian Medical University, 20 Cha-Zhong Road, Fuzhou, 350005 Fujian China; 2 Department of Radiology, Zhongshan Hospital Affiliated to Xiamen University, Xiamen, 361004 Fujian China; 3 Department of Biomedical Sciences, School of Veterinary Medicine, University of Pennsylvania, Philadelphia, PA 19104 USA

**Keywords:** Primary central nervous system lymphomas, diffusion, Perfusion

## Abstract

**Background:**

Diffusion and perfusion MRI can invasively define physical properties and angiogenic features of tumors, and guide the individual treatment. The purpose of this study was to investigate whether the diffusion and perfusion MRI parameters of primary central nervous system lymphomas (PCNSLs) are related to the tumor locations.

**Methods:**

We retrospectively reviewed the diffusion, perfusion, and conventional MRI of 68 patients with PCNSLs at different locations (group 1: cortical gray matter, group 2: white matter, group 3: deep gray matter). Relative maximum cerebral blood volume (rCBV_max_) from perfusion MRI, minimum apparent diffusion coefficients (ADC_min_) from DWI of each group were calculated and compared by one-way ANOVA test. In addition, we compared the mean apparent diffusion coefficients (ADC_mean_) in three different regions of control group.

**Results:**

The rCBV_max_ of PCNSLs yielded the lowest value in the white matter group, and the highest value in the cortical gray matter group (*P* < 0.001). However, the ADC_min_ of each subgroup was not statistically different. The ADC_mean_ of each subgroup in control group was not statistically different.

**Conclusion:**

Our study confirms that rCBV_max_ of PCNSLs are related to the tumor location, and provide simple but effective information for guiding the clinical practice of PCNSLs.

## Background

Primary central nervous lymphomas (PCNSLs) are malignant tumors with increased incidence rates [1]. To date, methotrexate-based chemotherapy is the cornerstone for PCNSLs treatment. The response to initial high-dose chemotherapy is a reliable indicator of the patients’ long-term survival [2–5]. As proposed by a previous study, the chemotherapy response is predominantly determined by the delivery of drugs through the vascular system [6]. Besides, some researchers have documented that PCNSLs with different cellular growth patterns may have different prognoses [7, 8].

Diffusion-weighted imaging (DWI) can assess the Brownian movement of water in the microscopic tissue environment by using apparent diffusion coefficient (ADC) values. Densely packed tumor cells with a high nuclear-to-cytoplasmic ratio could reduce water molecule motion [9, 10]. Few studies have suggested a significant correlation between ADC values and prognosis of patients with PCNSLs treated with methotrexate [11–13].

Dynamic susceptibility contrast perfusion-weighted imaging (DSC-PWI) can provide physiological information about vascular endothelial proliferation and angiogenesis [14–17]. Since tumor aggressiveness correlates with neovascularization, DSC-PWI is useful in the preoperative evaluation of brain tumors (including PCNSLs). Regional CBV (rCBV) derived from DSC-PWI can provide important hemodynamic information by intravenous injection of gadolinium-based contrast agent [18–20]. The response to chemotherapy is mainly determined by the delivery of drugs to the tumor through the vascular system [6]. Therefore, the rCBV value might be used as a biomarker of treatment responses in PCNSLs.

From the imaging perspective, diffusion and perfusion MRI may be helpful for a comprehensive assessment of the cellularity and vascularity, and a prediction of treatment response of PCNSLs. To our knowledge, there is no study in the literature combining diffusion and perfusion techniques to evaluate the PCNSLs of different locations. Therefore, our research aims to investigate whether DWI and DSC-PWI parameters are correlated with tumor locations, and provide evidence for clinical decision-making and prognostic evaluation.

## Methods

### Patients

The institutional review board of our hospital approved this study and informed consent was waived due to the retrospective design. Our institution’s database identified 166 patients who underwent MR examination for potential PCNSLs between October 2009 and September 2017. The inclusion criteria were as follow: (a) immunocompetent patients, (b) histopathological diagnosis of PCNSLs, (c) available pretreatment conventional MRI (cMRI), DWI and DSC-PWI. Patients with poor image quality were excluded. Ultimately, 68 patients (31 males and 37 females, age range 29–78 years; mean age 56.80 years) were included.

### MR imaging techniques

All MR images were acquired in the routine clinical workup using a 3.0 Tesla MRI system (Magnetom Verio TIM; Siemens Healthcare, Erlangen, Germany) with an eight-channel head coil. The cMRI protocols consisted of axial T1WI (TR/TE, 250 /2.48 ms), axial T2WI (TR/TE, 4000 /96 ms), axial FLAIR (TR, 9000 ms; TE, 94 ms; TI, 2500 ms), and contrast-enhanced T1WI (CE-T1WI; TR/TE, 250 ms/2.48 ms) in 3 orthogonal planes. It was uniform in all series about FOV 220 × 220 mm, section thickness 5 mm, and intersection gap 1.0 mm.

DWI was performed in the axial plane with a spin-echo-planar sequence (TR/TE, 8200/102 ms; NEX, 2.0; b-values, 0 and 1000 s/mm2). Corresponding ADC maps were generated automatically by the MRI system.

DSC-PWI was achieved with a gradient-recalled T2*-weighted echo-planar imaging sequence (TR/TE, 1000–1250/54 ms; NEX, 1.0; flip angle, 35°). In the first three phases, non-enhanced images were scanned to establish a pre-contrast baseline. When the scan was to the fourth phase of DSC-PWI, a standard dose of 0.1 mmol/kg of gadobente dimeglumine (Gd-BOPTA) was injected intravenously with a flow rate of 3 ml/s, followed by a 20 ml continuous saline flush.

### Data processing

All imaging assessments were performed on a Siemens workstation with standard software. All cMRI data concerning notch sign and contrast-enhancement pattern were assessed by two neuroradiologists who were blinded to tumor histology. When two observers disagreed, a senior neuroradiologist made the final decision.

To assess DWI data, ADC values were measured by manually placing ROIs on the ADC maps. At least five small round ROIs (25–40 mm^2^) were selected inside the tumor areas of visually lowest ADC. The ROI placements were made from the enhancing solid portion of the lesion, avoiding necrotic, cystic, hemorrhagic, or visible blood vessel that might affect the ADC values. For each patient, the enhancing solid portion of the tumor was identified on T2WI and CE-T1WI. Finally, the minimum ADC (ADC_min_) was calculated from the ROI with the lowest ADC value. The mean ADC (ADC_mean_) values of normal cortical gray matter (CGM), white matter (WM), and deep gray matter (DGM) were also calculated. ADC values were expressed as × 10^− 3^ mm^2^/s.

To evaluate DSC-PWI data, whole-brain CBV maps were generated using a single-compartment model. The relative maximum CBV value (rCBV_max_) was calculated by dividing the maximum CBV value of the tumor by the mean CBV value of the contralateral unaffected white matter. Therefore, the rCBV value was used as a quantitative parameter without unit. To minimize variances in rCBV_max_ values in each patient, measurements of rCBV_max_ values were performed with the same ROIs as those used for ADC measurement.

All the parameters derived from DWI and DSC-PWI were measured by a neuroradiologist (Z.X. with 9 years of experience in brain imaging) who blinded to the tumor histology.

### Statistical analysis

All quantitative parameters are presented as the means ± standard deviation (SD). A one-way ANOVA test was performed for the ADC_min_, ADC_mean_ and rCBV_max_ values with a Least Significant Difference (LSD) among groups. All *P-*values < 0.05 were considered to represent statistical significance. Statistical analysis was performed using SPSS software (Version 22.0, SPSS Inc., Chicago, USA) and MedCalc (Version 12.1.0, MedCalc Inc., Mariakierke, Belgium).

## Results

There was a total of 68 patients with 95 lesions. Totally 73 lesions (single lesion in 36 cases and multiple lesions in 17 cases) underwent DSC-PWI and 82 lesions (single lesion in 37 cases and multiple lesions in 23 cases) underwent DWI. The clinical and cMRI characteristics were summarized in Table 1. Homogenous contrast-enhancement pattern and notch sign were non-specific observed in PCNSLs regardless of location.

**Table 1:** The main clinical and cMRI features of three groups of PCNSLs

	CGM	WM	DGM	*P*
Sex (male/female)	15/17	7/15	9/5	.159
Age (year)	56.09 ± 9.8	57.96 ± 12.53	52.50 ± 13.83	.393
Contrast-enhancement pattern				.234
Homogeneous	29	30	19	
Heterogeneous	10	5	2	
Notch sign				.657
Yes	33	31	17	
No	6	4	4	

*CGM* Cortical gray matter; *WM* White matter; *DGM* Deep gray matter

**Fig. 1:**
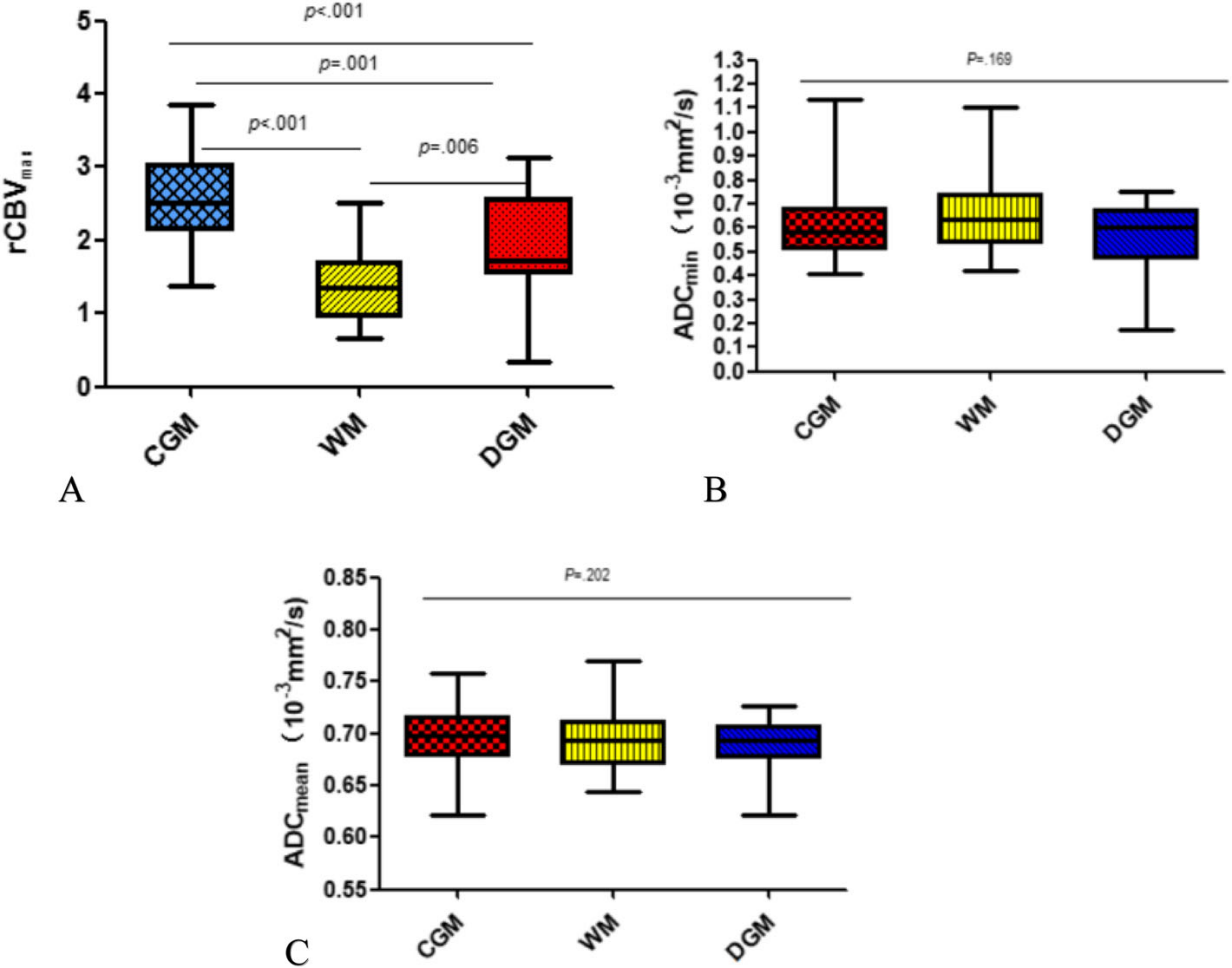
Comparison of (**a**) relative maximum cerebral blood volume (rCBV_max_) and(**b**) minimum apparent diffusion coefficient (ADC_min_) among PCNSLs of different locations of cortical gray matter (CGM), white matter (WM); deep gray matter (DGM), and (**c**) mean apparent diffusion coefficient (ADC_mean_) among control group of three different locations

**Fig. 2:**
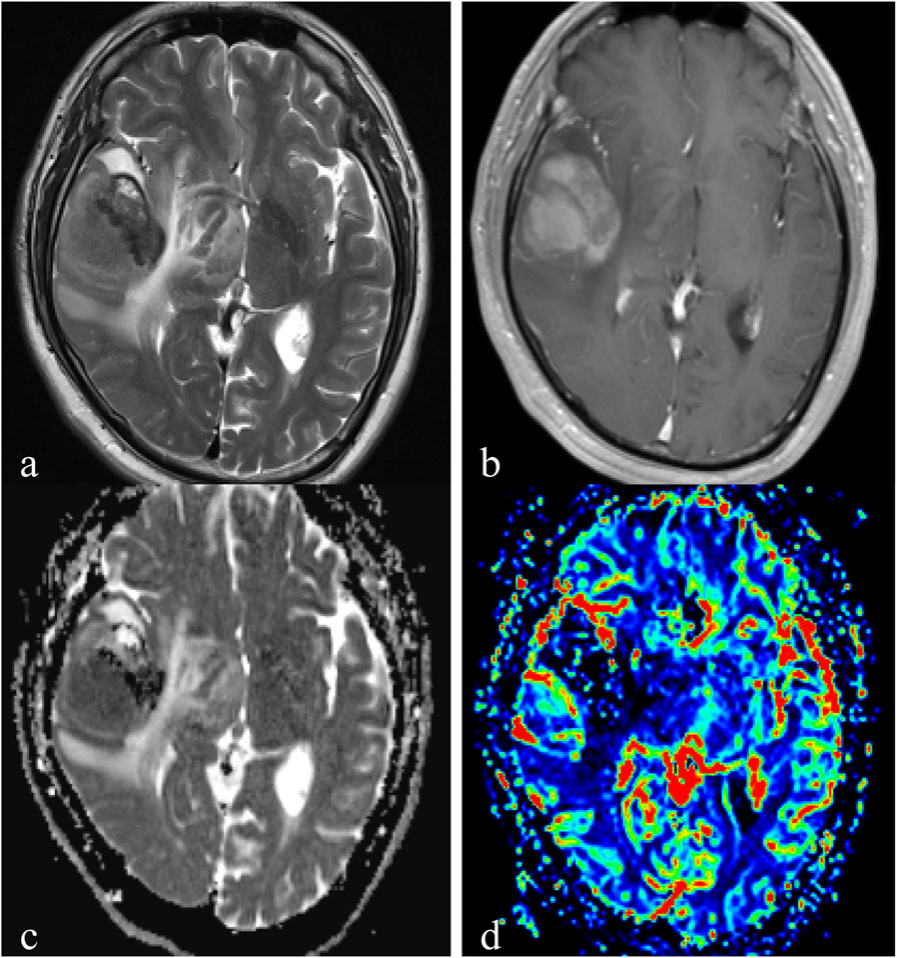
A 52-year-old man with a primary central nervous system lymphoma in the cortical gray matter. **a**. Axial T2WI demonstrates the heterogeneous high signal intensity on the right temporal lobe. **b**. Contrast-enhanced axial T1WI demonstrates a lesion enhancement. **c**. A corresponding ADC map shows the tumor with a decreased ADC value (ADC_min_ = 0.56 × 10^− 3^ mm^2^/s). The ADC_mean_ value is 0.68 × 10^− 3^ mm^2^/s in the left normal cortical gray matter. **d**. A correlative color CBV image shows highly elevated perfusion with the calculated rCBV_max_ of 3.42

**Fig. 3:**
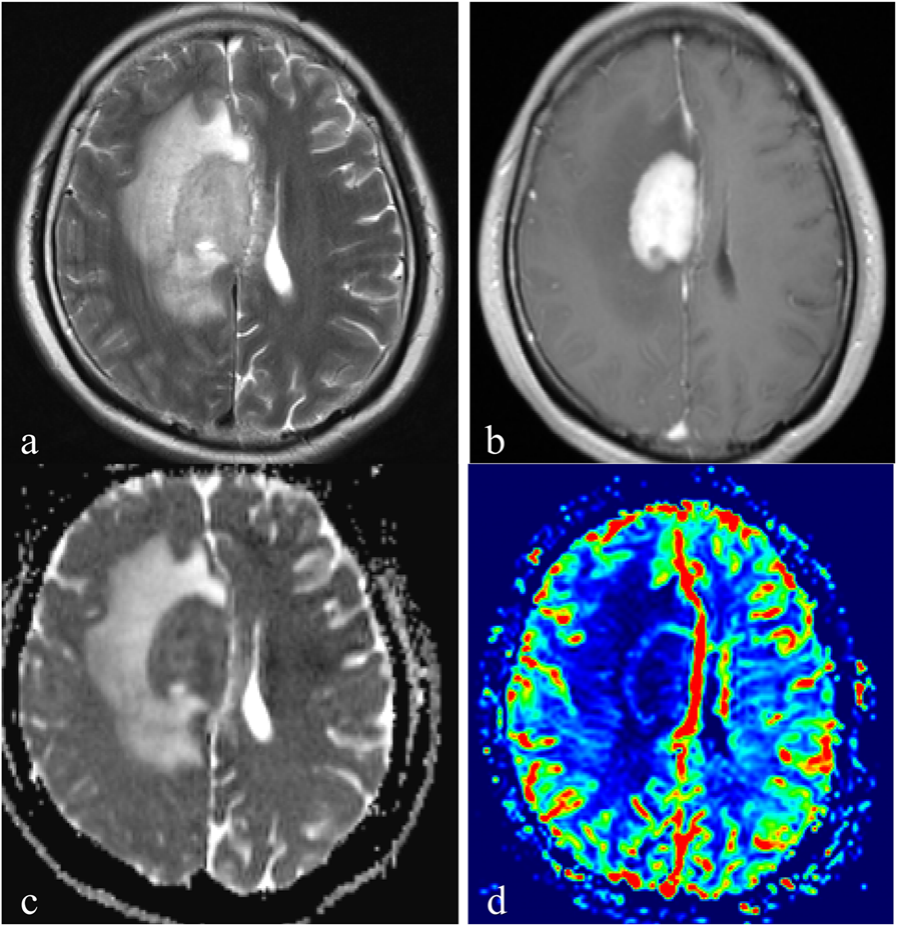
A 45-year-old woman with PCNSLs in the white matter. **a**. Axial T2WI shows the homogeneous high signal intensity on the right centrum semiovale. b. Contrast-enhanced axial T1WI demonstrates a lesion enhancement. **c**. A corresponding ADC map shows the tumor with a decreased ADC value (ADC_min_ = 0.55 × 10^− 3^ mm^2^/s). The ADC_mean_ value is 0.66 × 10^− 3^ mm^2^/s in the left normal white matter. **d**. A correlative color CBV image shows slightly elevated perfusion with the calculated rCBV_max_ of 0.76

**Fig. 4:**
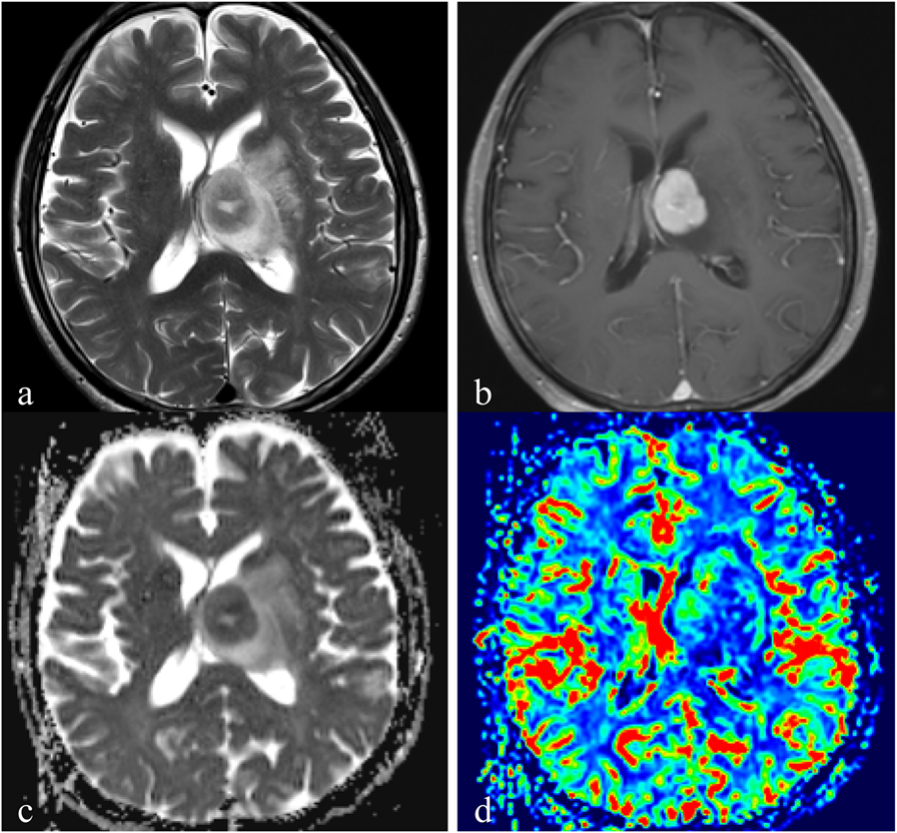
A 72-year-old man with PCNSLs in the deep gray matter. **a**. Axial T2WI demonstrates homogeneous high signal intensity on the left basal ganglia region. **b**. Contrast-enhanced axial T1WI demonstrates a lesion enhancement. **c**. A corresponding ADC map shows the tumor with a decreased ADC value (ADC_min_ = 0.49 × 10^− 3^ mm^2^/s). The ADC_mean_ value is 0.62 × 10^− 3^ mm^2^/s in the right normal deep gray matter. **d**. A correlative color CBV image shows moderately elevated perfusion with the calculated rCBV_max_ of 2.10

Table 2 and Fig. 1 showed the results of rCBV_max,_ ADC_mean_ and ADC_min_ for the three groups. A significant difference of rCBV_max_ was found among the three locations (*P* < 0.001). As shown in Figs. 1, 2, 3, 4, the rCBV_max_ values were lowest in WM and highest in CGM. However, ADC_min_ values of subgroup tumors were not significantly different (Fig. 1). In addition, ADC_mean_ values of subgroup of the normal control were not significantly different (Fig. 1).

**Table 2:** Comparison of DWI and DSC-PWI variables among the three groups of PCNSLs, and DWI variables among the three groups of control group

	CGM	WM	DGM	*P*
ADC_min_ (10^− 3^ mm^2^/s)	0.61 ± 0.15	0.65 ± 0.15	0.57 ± 0.14	.169
ADC_mean_ (10^− 3^ mm^2^/s)	0.70 ± 0.03	0.69 ± 0.03	0.69 ± 0.02	.202
rCBV_max_	2.55 ± 0.64	1.34 ± 0.46	1.87 ± 0.74	<.001

*CGM* Cortical gray matter; *WM* White matter; *DGM* Deep gray matter

## Discussion

In our study, we present the evidence that rCBV_max_ values of PCNSLs are related to their locations. The CGM PCNSLs have the highest rCBV_max_, and the WM tumors have the lowest rCBV_max_. However, there were no significant differences among ADC_min_ values of PCNSLs with different locations.

The role of cMRI in the characterization of PCNSLs has been well established [21]. However, the cMRI features of PCNSLs (homogenous contrast-enhancement pattern and notch sign) at various sites are non-specific.

ADC_min_ values have been extensively used to investigate PCNSL and its prognosis [13, 22, 23]. Previous studies have shown a significant inverse correlation between tumor cell density and ADC value in PCNSLs, suggesting ADC to be a surrogate marker for tumor proliferation and Ki-67 index [23–25]. Barajas et al. [11] reported that ADC measurements could predict clinical prognosis in patients with PCNSLs. The study indicated that lower ADC_25%_ and ADC_min_ values were predictive of shorter progression-free survival (PFS) and overall survival (OS). Similarly, Valles et al. [26] found that both mean and minimum ADC values of PCNSLs have strong correlations with patients’ PFS and OS. However, as shown by Schob et al., proliferative activity revealed by ADC was not related to the location of PCNSLs [23]. In accordance with the previous study, our research demonstrated no correlations between ADC_min_ and the locations of PCNSLs. Meanwhile, our research also confirmed that there was no differences in ADC_mean_ values between different locations of control group. Therefore, it is conceivable that DWI variables may not be varied among PCNSLs with different locations.

DSC-PWI has the potential to provide hemodynamic information about PCNSLs [27–30]. In healthy subjects, the CGM structures have the highest blood volume, followed by DGM and WM [31]. PCNSLs represent unique angiocentric growth patterns, but lack abundant neoangiogenesis [1, 32, 33]. Thus, we can infer that the PCNSLs of CGM have the highest rCBV, followed by DGM and WM tumors. Our findings are in good agreement with such a theoretical hypothesis. PCNSLs are usually treated by high-dose methotrexate or radiation therapy [5, 34]. The response to initial methotrexate therapy is significantly associated with patient’s prognosis [35]. Clinicians could adjust the drug dose and initiate individualized second-line salvage therapies in chemotherapy-resistant patients, which may minimize neurological toxicity and improve prognosis [36]. It is commonly acknowledged that the delivery of the drug through the vascular system determines the response to chemotherapy [6]. Notably, Valles et al. [26] reported that rCBV value could be a valid imaging biomarker of clinical outcome: patients of PCNSLs with lower tumor rCBV values at pre-therapy baseline have significantly shorter PFS and OS compared with the control group. Therefore, we can also postulate that PCNSLs of CGM have the best chemotherapeutic response and clinical prognosis, followed by DWM and WM tumors. Location of PCNSLs could stratifies the patients into different risk groups to guide the methotrexate-based chemotherapy.

There are some potential limitations in our study. First, we did not verify the clinical prognostic factors of PCNSLs, although Valles et al. [26] confirmed that tumor rCBV values might be biomarkers of treatment response. A multicentered prospective investigation with long-term follow-up should be further studied to verify our speculation. Second, this retrospective study may lead to some biases in the case selection. Therefore, more extensive trials are needed to validate and establish expected datasets to refine current scores and establish new risk factors. Third, due to the lack of available tumor-tissue samples, we were unable to establish a point to point rCBV_max_ measurements associated with histopathological characteristics.

## Conclusion

Our study confirms that the cerebral blood supplies of the PCNSLs are related to their locations. We present the first evidence of tumor location as a simple but effective prognostic indicator of PCNSLs. The PCNSLs of CGM may have the best prognosis, whereas tumors of WM may have the worst prognosis.

## Data Availability

Due to statutory provisions regarding data and privacy protection, the dataset supporting the conclusions of this article is available upon individual request directed to the corresponding author.

## Abbreviations

MRI: Magnetic resonance imaging
PCNSLs: Primary central nervous system lymphomas
rCBV_max_: Relative maximum cerebral blood volume
rCBV: Relative cerebral blood volume
CBV: Cerebral blood volume
ADC_min_: Minimum apparent diffusion coefficients
DWI: Diffusion weighted imaging
ADC_mean_: Mean apparent diffusion coefficients
ADC: Apparent diffusion coefficients
DSC-PWI: Dynamic susceptibility contrast perfusion-weighted imaging
cMRI: Conventional magnetic resonance imaging
T1WI: T1 weighted imaging
T2WI: T2 weighted imaging
FLAIR: Fluid attenuated inversion recovery
TR: Repetition time
TE: Echo time
TI: Inversion time
CE-T1WI: Contrast-enhanced T1 weighted imaging
Gd-BOPTA: Gadobente dimeglumine
FOV: Field of view
NEX: Number of excitation
ROI: Region of interest
CGM: Cortical gray matter
WM: White matter
DGM: Deep gray matter
SD: Standard deviation
LSD: Least significant difference
OS: Overall survival
PFS: Progression-free survival
